# Stress, hypothalamic-pituitary-adrenal axis, hypothalamic-pituitary-gonadal axis, and aggression

**DOI:** 10.1007/s11011-024-01393-w

**Published:** 2024-07-31

**Authors:** Ngala Elvis Mbiydzenyuy, Lihle-Appiah Qulu

**Affiliations:** 1 Basic Science Department, School of Medicine, Copperbelt University, P.O Box 71191, Ndola, Zambia; 2 Division of Medical Physiology, Biomedical Science Research Institute, Stellenbosch University, Private Bag X1, Matieland, 7602 Cape Town South Africa

**Keywords:** Androgen system, Aggression-related disorders, Glucocorticoids, NeurobiologyTestosterone

## Abstract

This comprehensive review explores the intricate relationship between the hypothalamic-pituitary-adrenal (HPA) axis, the hypothalamic-pituitary-gonadal (HPG) axis, and aggression. It provides a detailed overview of the physiology and functioning of these axes, as well as the implications for aggressive behavior. The HPA axis, responsible for the stress response, is activated in response to various stressors and can influence aggressive behavior. Glucocorticoids, such as cortisol, play a crucial role in stress-induced activation of the HPA axis and have been implicated in aggressive tendencies. Chronic stress can dysregulate the HPA axis, leading to alterations in cortisol levels and potentially contributing to aggressive behavior. The HPG axis, particularly the androgen hormone testosterone, is also closely linked to aggression. Animal and human studies have consistently shown a positive association between testosterone levels and aggression. The androgen receptors in the brain’s neural circuitry play a critical role in modulating aggressive behavior. Interactions between the HPA and HPG axes further contribute to the regulation of aggression. Feedback mechanisms and crosstalk between these axes provide a complex system for the modulation of both stress and reproductive functions, which can impact aggressive behavior. Additionally,the influence of stress on reproductive functions, particularly the role of androgens in stress-induced aggression, adds further complexity to this relationship. The review also discusses the future directions and implications for clinical interventions. Understanding the neurobiological mechanisms underlying aggression requires integrating molecular, cellular, and circuit-level approaches. Translational perspectives, including animal models and human studies, can bridge the gap between basic research and clinical applications. Finally, therapeutic strategies for aggression-related disorders are explored, highlighting the importance of targeted interventions based on a comprehensive understanding of the interactions between the HPA and HPG axes. In conclusion, this review provides a comprehensive overview of the physiological and neurobiological mechanisms underlying aggression, with a specific focus on the interplay between the HPA and HPG axes. By elucidating the complex interactions between stress, hormones, and aggressive behavior, this research paves the way for future investigations and potential therapeutic interventions for aggression-related disorders.

## Introduction

Stress is defined as “the non-specific response of the body to any demand for change” (Selye 1936). It can be described as the effect of anything, real or potential, that threatens the constancy of the internal environment. A stressor is a stimulus that disrupts homeostasis and activates a variety of chemical and specific physiological processes and behavioral coping mechanisms to restore homeostasis and promote survival (Cizauskas et al. 2015). Stressors are broadly categorized into physical (or physiological) and psychological stressors. Physical stressors refer to biological agents (e.g., bacteria, viruses) or external forces (e.g., radiation, noise) that can modify exposure and/or elicit a physiological response from the exposed organism (Rider et al. 2014). Psychological stressors are social and physical environmental circumstances that challenge the adaptive capabilities and resources of an organism (Chiappelli et al. 2024). In the face of real or potential threats, interconnected systems release mediating molecules, which bind to their respective receptors in the brain and periphery to bring about the stress response, which through physiological and behavioral mechanisms restores the body homeostasis and promotes adaptation (Ayres 2020). These interactions comprise components of nervous system, including the hypothalamic-pituitary-adrenal (HPA) axis, the sympathetic-adreno-medullary (SAM) axis, the endocrine, immune systems and other diffuse systems in the brain, such as the locus coeruleus-norepinephrine system (Kopin et al. 1988; McCarty et al. 1988). The activation of these systems helps prepare the body to deal with psychologically stressful conditions (Won and Kim 2016). The set of non-specific responses constitute what is called the General Adaptation Syndrome first described by Selye and develops in three stages: (1) alarm phase, characterized by acute manifestations such as lowered body temperature and blood pressure, and muscle tone is decreased; (2) resistance phase, when the acute manifestations disappear; and (3) exhaustion phase, when first stage reaction may be present again or when it may cause the collapse of the organs (Selye 1936).

Aggression is a complex phenomenon exhibited by virtually all animal species as a form of social communication with the aim of territorial control. This is often characterized in extreme cases by behaviors that may inflict harm to the opponent as well as to the aggressor. Aggression has been classified in a variety of ways, to include offensive aggression (which is a form of aggressive behavior characterized by initiative of the offender and a range of introductory, often threatening, behavioral displays before attempting to reach the back and neck as non-vulnerable targets for the consummatory aggressive attack bites) (Koolhaas et al. 2013), defensive aggression (aggressive behavior performed in response to an attack by another individual such as a dangerous or evasive response to a sense of fear), and parental aggression (e.g., maternal aggression) (Denson et al. 2018; McCarty et al. 1988). Examples exist of functional aggression turning into violence, which can thus be defined as an injurious form of offensive aggression that is out of control and out of context (Neumann et al. 2010). It is a pathological form of offensive behaviour that is no longer subject to inhibitory control mechanisms and that has no additive functional value to normal aggressive behaviour in social communication (Blanchard et al. 1977; Koolhaas et al. 2013). Violence in animals has been described as an escalated, pathological and abnormal form of aggression characterized primarily by early attack latencies, prolonged and frequent consummate behaviors and attack bites (Miczek et al. 2003). Sexual aggression is a form of violent aggression representing a continuum of illicit behaviours characterized by any unwanted or non-consensual sexual activity (Hales and Gannon 2019). The World Health Organisation defines it as any sexual act, or an attempt to obtain such, unsolicited sexual innuendoes and comments, or use of force to obtain sexual pleasure, by any person irrespective of their affiliation to the victim in any setting, including but not limited to home and work (World Health Organization 2011).

It has been suggested hypothetically that traumatic stress is at the root of sexual aggression (Nordman et al. 2020). A suggested mechanism of aggression is the imbalance or altered balance between the inhibitory and excitatory stimuli from cortex to the sub-cortical and limbic structures (Kelley et al. 2019). This imbalance results in inadequate risk assessment and top-down inhibition by the prefrontal cortex (Ong et al. 2019). Bottom up outputs that have increased frequency and amplitude especially from the amygdala (largely associated with aggressive behavior) toward the orbitofrontal cortex contribute to impulsive aggression (Wright et al. 2008). Real or potential threats hyperactivate the amygdala and other limbic structures and inhibit top-down control systems in the prefrontal cortex. Thus, an imbalance between prefrontal regulatory influences and hyper-responsivity of the amygdala and other limbic regions has been implicated in the modulation of aggressive behavior (Siever 2008). In many human and animal studies, ventral prefrontal cortex (vPFC) has been shown to be associated aggression. This area receives inputs from various brain areas including stimuli from hypothalamic nuclei (Lickley and Sebastian 2018).

While researchers accept that aggression could be a form of behavioral response to social, environmental, or biological stressors, aggression is potentially harmful, stressful, and traumatic. This suggests that the neurobiological mechanisms responsible for aggressive behavior should include in greater extents biological mechanisms involved in stress responses, as hyper-arousal-driven aggressiveness, is related to acute exaggerated glucocorticoid response to stress, and can be seen in conditions such as post-traumatic stress disorder (Vaeroy et al. 2019). The evolutionarily-conserved and complex systems that are responsible for the stress response and modulated at several levels of the central nervous system (CNS), may relate, interact or activate systems that mediate aggressive behavior (Aikins et al. 2021; Senst and Bains 2014). The stress response has been shown to involve activity in the hypothalamic–pituitary axis (HPA axis), but how this interacts with the HPG axis, to aggressive behavior is still unclear. There has however been substantial progress in our understanding of the neurobiology of stress from animal and human studies (Bock et al. 2015; Campagne 2019; KG et al. 2016; Loi et al. 2017; Murison 2016). It is important to provide recent perspectives of the relationship between stress and aggressive behavior.

## The HPA axis: physiology and implications in aggression

### HPA axis components and functioning

The hypothalamic-pituitary-adrenal (HPA) axis is a brain pathway that is centrally involved in regulating the body’s response to stress. Magnocellular neurons in the medial parvocellular division of the paraventricular nuclei (PVN) of the hypothalamus, synthesize and secrete corticotropin-releasing factor (CRF), the main regulator of the HPA axis (Jiang et al. 2019). Signals from the limbic areas and prefrontal cortex also stimulate the secretion of arginine vasopressin (AVP) from the paraventricular nucleus which is co-stored with CRF and released into the hypophyseal portal system in the median eminence where it binds to its receptor, CRF type1 receptor (CRF1) on the anterior pituitary gland, causing it to release adrenocorticotropic hormone (ACTH) into the systemic circulation (Vasconcelos et al. 2020). In addition to the corticotropes on the anterior pituitary gland, CRF1 is expressed in high density in the cerebral cortex, cerebellum, hippocampus and amygdala; its peripheral expression is less robust and concentrated to skin, blood vessels, skin, lung, testes, ovaries and placenta (Binder and Nemeroff 2009). CRF type 2 receptor (CRF2) is expressed primarily in peripheral tissues including skeletal muscles, gastrointestinal tract, and heart, as well as in subcortical structures of the brain including the hypothalamus, pituitary, amygdala, bed nucleus of the stria terminalis and raphe nuclei (Godoy et al. 2018). A binding protein expressed in the pituitary gland, placenta, liver and other brain parts called cortisol releasing hormone binding protein (CRF-BP) binds with CRF to CRF receptors with a higher affinity than CRF (Vandael and Gounko 2019). Studies show that 40–60% of CRF is bound to CRF-BP and its role also is to regulate the bioavailability of CRF (Westphal and Seasholtz 2005). Expression of this binding protein increases in response to stress, and plays a feedback role in decreasing the interaction of CRF with CRF type 1 receptors (Ketchesin et al. 2017).

**Fig. 1:**
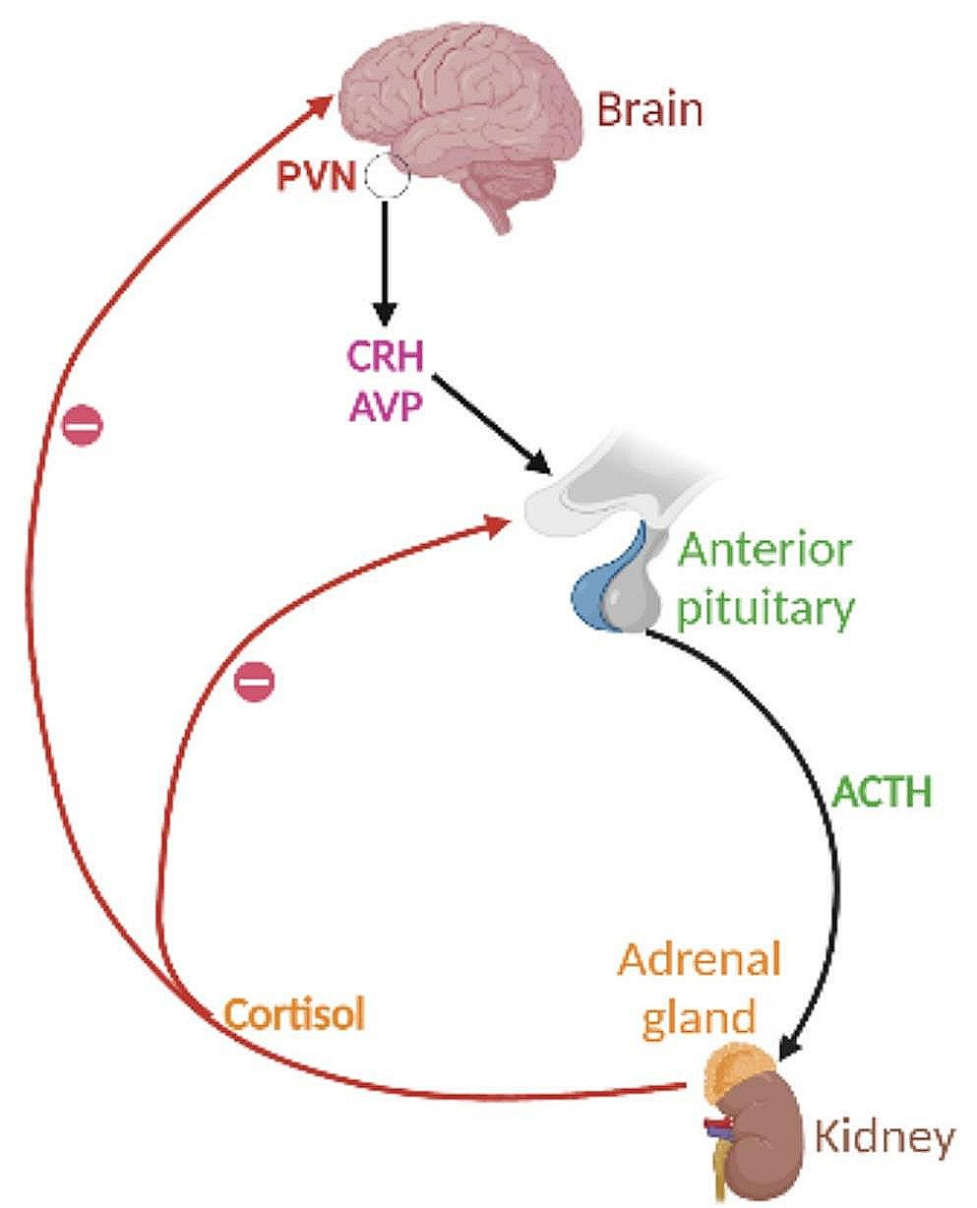
Hypothalamic-pituitary-adrenal (HPA) axis schematic. The paraventricular nucleus of the hypothalamus (PVN) releases arginine vasopressin (AVP) and corticotropin-releasing hormone (CRH) in response to perceived stress. AVP is then transferred to the anterior pituitary, where it triggers the release of adrenocorticotropic hormone (ACTH) into the bloodstream. The production of glucocorticoids (CORT), which have a variety of physiological consequences, is induced by ACTH in the adrenal cortex. In order to reduce excessive activation of the HPA axis, glucocorticoids also exert negative feedback at the level of the brain and pituitary

**Fig. 2:**
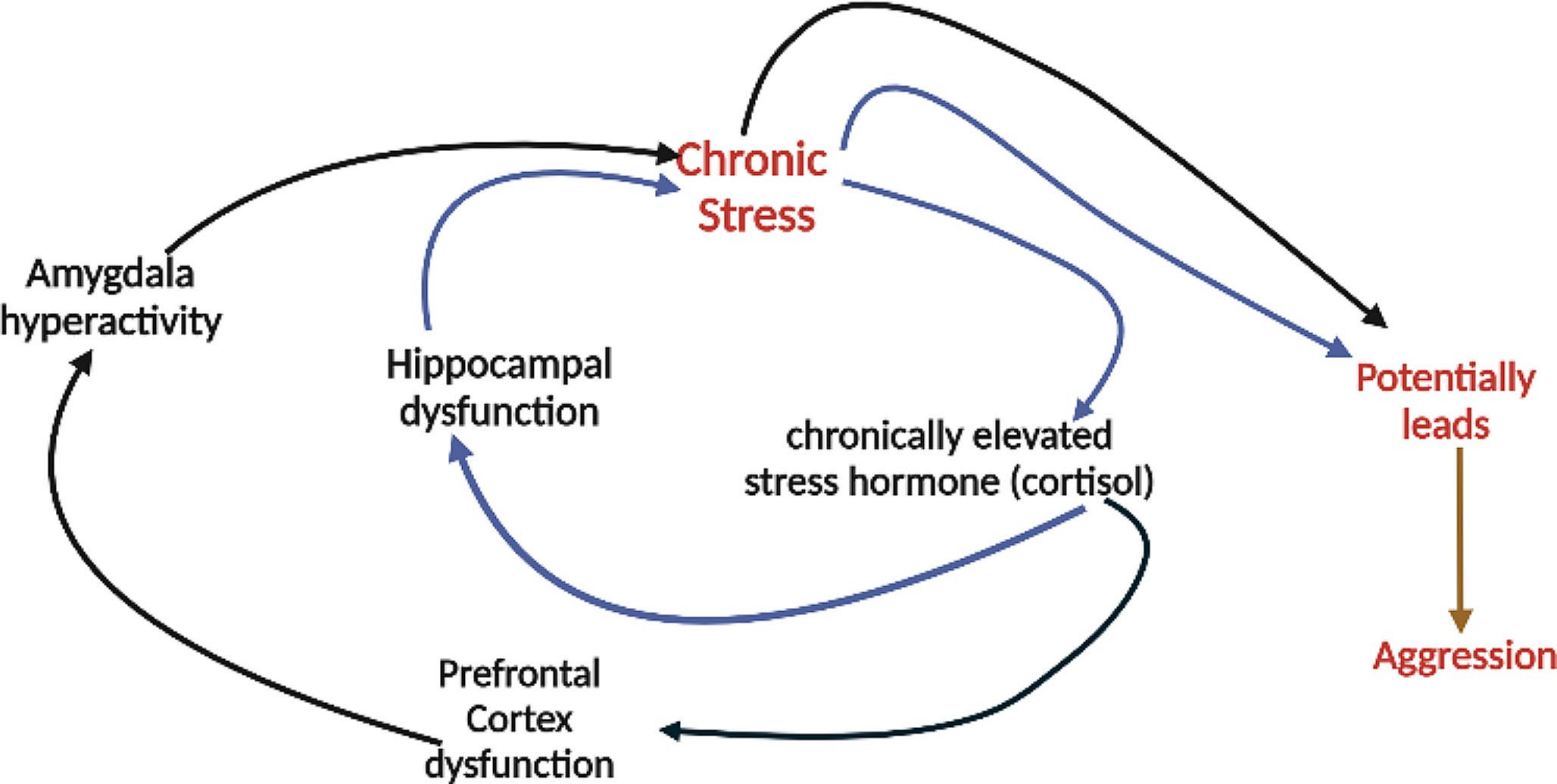
The schematic illustrates the interplay between chronic stress, the hypothalamic-pituitary-adrenal (HPA) axis, the prefrontal cortex, the amygdala, and the hippocampus in their roles in aggression. It depicts the bidirectional relationships and regulatory mechanisms among these components. Chronic stress activates the HPA axis, leading to increased release of stress hormones, such as cortisol. Elevated cortisol levels can have detrimental effects on the prefrontal cortex, impairing its regulatory control over emotions and aggression. The prefrontal cortex, responsible for executive functions and emotional regulation, has inhibitory connections with the amygdala, a brain region involved in emotional processing and aggression. Chronic stress and dysregulation of the HPA axis can disrupt this inhibitory control, resulting in increased amygdala activation and heightened aggression. The hippocampus, crucial for memory formation and stress regulation, interacts with the HPA axis and can be affected by chronic stress. Dysfunction in the hippocampus can further contribute to dysregulation of the stress response and exacerbate aggressive behavior. Overall, the schematic provides a visual representation of the intricate relationships between chronic stress, the HPA axis, the prefrontal cortex, the amygdala, and the hippocampus in shaping aggression

**Fig. 3:**
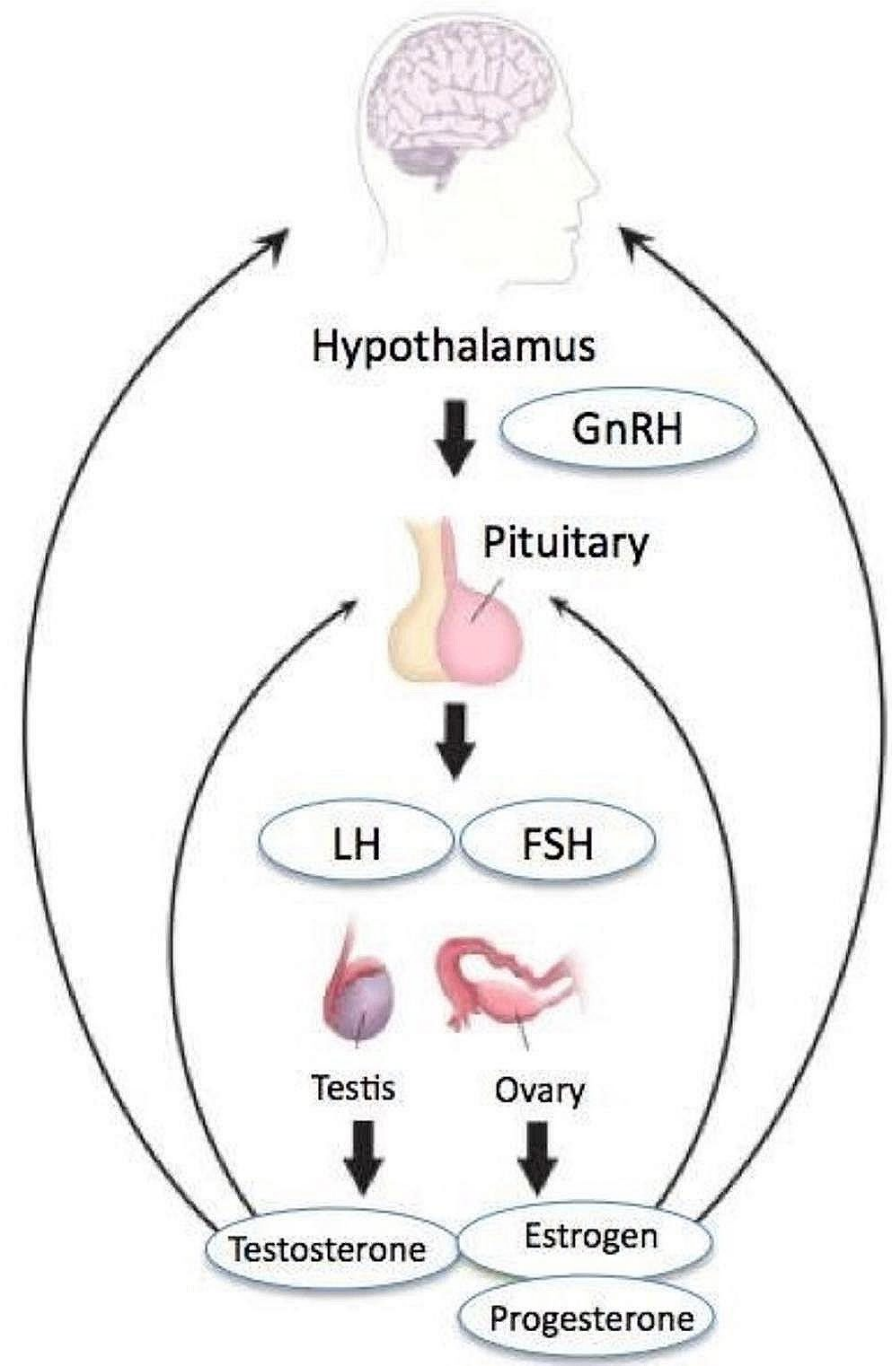
The schematic illustrates the hypothalamic-pituitary-gonadal (HPG) axis in both males and females. It highlights the key components and interactions involved in regulating reproductive functions. In females, the hypothalamus releases gonadotropin-releasing hormone (GnRH), which stimulates the anterior pituitary gland to release follicle-stimulating hormone (FSH) and luteinizing hormone (LH). FSH acts on the ovaries to stimulate follicle development and estrogen production, while LH triggers ovulation and promotes progesterone synthesis. The ovaries, in turn, produce estrogen and progesterone, which regulate the menstrual cycle and support reproductive processes. In males, the hypothalamus also releases GnRH, which stimulates the anterior pituitary to release FSH and LH. FSH acts on the testes to promote sperm production (spermatogenesis), while LH stimulates Leydig cells in the testes to produce testosterone. Testosterone, the primary male sex hormone, plays a crucial role in male reproductive function, including the development of secondary sexual characteristics and the regulation of sexual desire. The schematic demonstrates the reciprocal feedback loops within the HPG axis, where hormones produced by the gonads exert negative feedback on the hypothalamus and pituitary gland to regulate hormone secretion. This feedback mechanism helps maintain hormonal balance and regulate reproductive functions

**Fig. 4:**
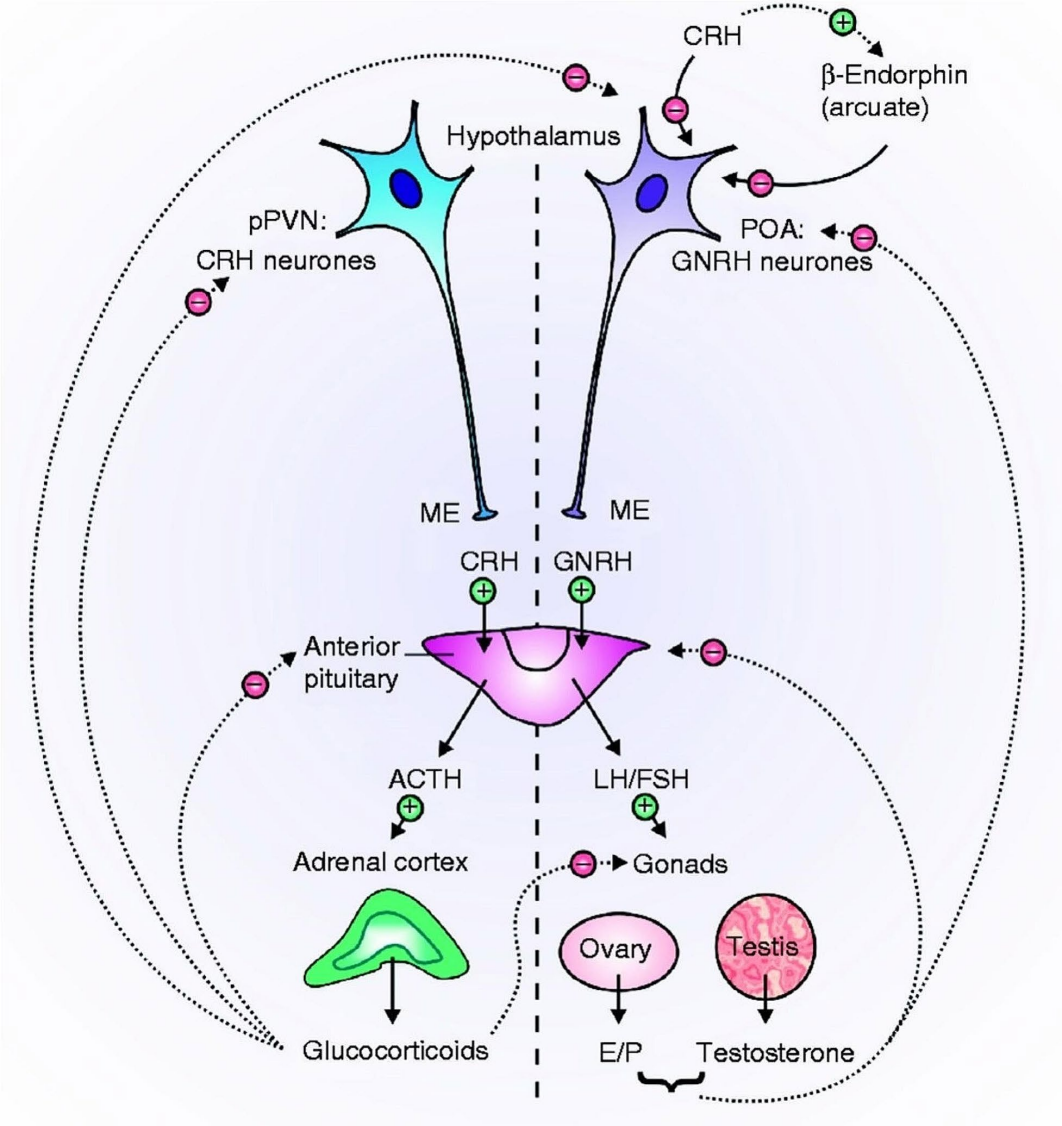
Interactions between the hypothalamic-pituitary-gonad (HPG) and HPA axes (Brunton 2013). HPG axis: From their terminals in the median eminence (ME), GNRH neurones in the preoptic region (POA) of the hypothalamus synthesize and release GNRH. GNRH stimulates (C) the production and release of LH and follicle-stimulating hormone (FSH) into the circulation by acting on gonadotrophs in the anterior pituitary gland. Leydig cells in the testis are stimulated by LH to produce testosterone, whereas FSH increases spermatogenesis. In the ovary, FSH governs follicular maturation and the generation of oestradiol (E 2), whereas LH controls ovulation and the corpus luteum’s secretion of progesterone. At the level of the hypothalamus and anterior pituitary, gonadal sex steroid hormones have negative feedback (K) effects. Stress exposure triggers CRH neurones in the parvocellular division of the paraventricular nucleus (pPVN) of the hypothalamus, which results in CRH release at the ME. This process is known as the HPA axis. The anterior pituitary is stimulated by CRH to release ACTH, which in turn activates the adrenal cortex to secrete glucocorticoids. Glucocorticoids influence the anterior pituitary and PVN through negative feedback. interactions between the HPA and HPG axes: glucocorticoids have a variety of effects on the HPG axis. Glucocorticoids prevent the production and release of GNRH in the hypothalamus and LH secretion in the anterior pituitary. Ovarian and testicular glucocorticoid receptors are present, and glucocorticoids work to prevent the production of gonadal steroids in these tissues. Stress-related increases in CRH levels also block GNRH neurones, either directly or indirectly, by stimulating b-endorphin in the arcuate nucleus

Arginine vasopressin also facilitates the release of ACTH by binding to its vasopressin V1b receptor on the corticotropes (Deng et al. 2017; Lee et al. 2015; Roper et al. 2011). The crucial role of the CRF in the activation of the HPA axis was demonstrated in a study in which deletion of the CRF gene showed blockage of ACTH release in both basal and stress conditions (Zhou and Fang 2018). The released ACTH binds to its receptor, a type 2 melanocortin receptor (MC2-R) on the adrenal cortex where it stimulates the synthesis and release of glucocorticoids (GC) into the general circulation (Hostinar et al. 2014). The adrenal cortex secretes approximately 20–30 mg of cortisol daily, reaching serum levels ranging from 5 to 24 µg/dL. However, under stress conditions, secretion may increase 10- to 12-fold (Bornstein et al. 2016). In circulation 90–95% of the cortisol is bound to plasma proteins, mostly to an alpha globulin called corticosteroid- binding globulin (CBG) or transcortin and a minority to albumin. The remaining 5–10% of the cortisol is free and is the physiologically active form (Vandael and Gounko 2019). These glucocorticoids exert the physiological effects of the HPA axis as well as feedback negatively to inhibit further activation of the axis (Tsigos and Chrousos 2002). This feedback inhibition by circulating glucocorticoids is an important mechanism for controlling HPA activity (Figs. 1, 2, 3, 4).

### Stress-induced activation of the HPA axis

The body’s response to stress involves activation of Hypothalamic-Pituitary-Adrenal (HPA) axis, which causes the release of corticotropin-releasing factor (CRF). CRF binds to its receptors on the anterior pituitary gland resulting in the release of adrenocorticotropic hormone (ACTH). The released ACTH binds to its receptors on the adrenal cortex and stimulates adrenal release of cortisol. Cortisol is released for several hours and at a certain blood concentration, it acts on various target tissues, including the brain, to modulate physiological and behavioral responses to stress. In response to stress-induced cortisol release, several physiological changes occur. Cortisol mobilizes energy stores, increasing blood glucose levels and providing the body with a readily available energy source. It also enhances cardiovascular function, preparing the body for a fight-or-flight response. Additionally, cortisol dampens the immune response to conserve energy for immediate survival needs. Circulating cortisol then exerts negative feedback on the hypothalamic release of CRF and the pituitary release of ACTH (negative feedback), resulting in systemic homeostasis.

While the activation of the HPA axis is crucial for coping with acute stressors, chronic or prolonged stress can dysregulate this system. Excessive or prolonged cortisol release can have detrimental effects on the body and brain. Chronic stress can lead to dysregulation of the HPA axis, resulting in alterations in cortisol secretion patterns. Individuals exposed to chronic stress may exhibit hyperactivity or blunted reactivity of the HPA axis. Hyperactivity refers to an exaggerated response of the HPA axis, with heightened cortisol levels even during non-stressful periods. On the other hand, blunted reactivity describes a diminished cortisol response to stressors. Both dysregulation patterns have been associated with negative health outcomes and increased vulnerability to various disorders, including depression, anxiety, and aggression.

The dysregulation of the HPA axis in response to chronic stress has significant implications for aggression. Stress-induced alterations in cortisol levels can disrupt the delicate balance of neurotransmitter systems involved in aggression regulation, such as serotonin and dopamine. Additionally, chronic stress can affect brain regions implicated in aggressive behavior, including the amygdala, prefrontal cortex, and limbic system, leading to changes in emotional processing, impulse control, and decision-making. Understanding the complex interplay between chronic stress, HPA axis dysregulation, and aggression is crucial for developing effective interventions. Targeted therapies aimed at restoring HPA axis balance and reducing chronic stress may hold promise in mitigating aggressive behaviors associated with dysregulated stress responses.

### Glucocorticoids and aggression

Glucocorticoid receptors are ubiquitously distributed throughout the brain (in both glial cells and neurons) and peripheral tissues, and are of two types: mineralocorticoid (type I) receptors and glucocorticoid (type II) receptors (Nicolaides et al. 2020). There is a high expression of Type I receptors in limbic areas like the entorhinal cortex and hippocampus, all implicated in memory formation and enhancement, while type II receptors are found in limbic areas (amygdala, hippocampus and hypothalamus) and cortical areas, most critically the prefrontal cortex (Viho et al. 2019). In prolonged stress, in which the levels of glucocorticoids are high, their increased activity may lead to atrophy of the hippocampus through interfering with brain structures and chemical processes that are implicated in memory formation and retrieval (Hawkley et al. 2012). In vivo and in vitro results showed that GC tend to inhibit/reduce hippocampal neurogenesis through activation of both types of receptors (Anacker et. Al. 2013). Some studies in rodents however suggest that predictable chronic mild stress (PCMS) such as the routine stress experienced in day-to-day life increases adult neurogenesis in the hippocampal dentate gyrus and improved mood and cognitive function (Kirby et al. 2013; Parihar et al. 2011; Surget and Belzung 2021). Chronic exposure to glucocorticoids preferentially activates type II receptors and induces neuronal death and attenuation of neurogenesis (Lupien et al. 1998). Researchers found that patients with Cushing’s syndrome (a condition in which there is abnormally high levels of the hormone cortisol) showed a reduction of the hippocampal volume that was accompanied with severe memory impairments. The hippocampal damage disrupts the normal inhibitory control on the HPA axis, leading to an excess of CRF secretion and eventually to increased glucocorticoid levels, worsening the hippocampal atrophy (Resmini et al. 2012). Changes in hippocampal structure and function have been implicated in several psychiatric disorders and behavioral abnormalities, including aggression. Multiple studies have demonstrated a negative correlation between hippocampal volume and aggressive behavior. Individuals with smaller hippocampal volumes have been found to exhibit higher levels of aggression compared to those with larger hippocampal volumes.

There is data suggesting that activation of the type I receptor is required for appropriate regulation of stress responses in some situations and certain immediate actions of glucocorticoid in the basolateral amygdala and hippocampus such as impairment of spatial memory retrieval and facilitate memory of emotionally arousing experiences, are mediated by type I receptor activation (Barsegyan et al. 2019; Faught and Vijayan 2018). While the link between inhibited neurogenesis and aggressive behavior is not clear, a study showed that repeated experience of winning in a social conflict heightens aggression, increases proliferation of neuronal progenitors and production of young neurons in the hippocampus, and decreases neuronal activity in the amygdala (Smagin et al. 2015). These suggest that alterations in hippocampal structure may contribute to the expression of aggressive tendencies. One proposed mechanism for the link between hippocampal volume and aggression involves the influence of the hippocampus on emotion regulation. The hippocampus is involved in the modulation of stress responses and plays a crucial role in inhibiting the hypothalamic-pituitary-adrenal (HPA) axis, which is responsible for the release of stress hormones. Dysfunction in this regulatory system can lead to heightened stress responses and increased aggression. Additionally, the hippocampus interacts with other brain regions, such as the amygdala, which is known to play a role in emotional processing and aggression. Disruptions in the communication between these regions may contribute to alterations in aggressive behavior.

Glucocorticoids exert their influence on aggression through intricate interactions with various neural circuits and neurotransmitter systems. These hormones can modulate the activity of brain regions involved in emotional processing and behavioral control, such as the amygdala, prefrontal cortex, and hippocampus. The amygdala, known for its role in the regulation of emotions, is particularly sensitive to glucocorticoid signaling. High levels of glucocorticoids have been associated with increased amygdala activity, leading to heightened emotional responses, including aggression. Moreover, the prefrontal cortex, involved in decision-making and impulse control, is influenced by glucocorticoids, and disruptions in prefrontal cortex functioning may contribute to impulsive and aggressive behaviors (Gjerstad et al. 2018; Koning et al. 2019). The relationship between glucocorticoids and aggression is complex and can be influenced by various factors. Acute stressors can temporarily increase glucocorticoid levels, resulting in heightened arousal and aggression. However, chronic stress and long-term exposure to elevated glucocorticoids have been associated with increased aggression. Prolonged activation of the stress response can lead to changes in the brain’s neurochemistry and structural alterations, which may contribute to the development of aggressive behaviors. Additionally, individual differences in glucocorticoid sensitivity and stress reactivity may contribute to variability in aggressive tendencies among individuals.

Animal studies have provided valuable insights into the role of glucocorticoids in aggression. Manipulations of glucocorticoid levels, such as administering exogenous glucocorticoids or blocking glucocorticoid receptors, have demonstrated their influence on aggressive behavior. For example, studies have shown that administering glucocorticoids can increase aggressive responses in animals, while blocking glucocorticoid receptors can reduce aggression (Dunlap et al. 2011; Notari et al. 2022). These findings suggest that glucocorticoid signaling is involved in the regulation of aggression. In humans, studies examining the relationship between glucocorticoids and aggression have yielded mixed results. Some studies have reported associations between higher cortisol levels and increased aggression, particularly in individuals with certain psychiatric disorders or in situations of chronic stress (McBurnett et al. 2000; Meruelo et al. 2023; Trifu et al. 2020). However, other studies have found no significant correlation or even inverse relationships between cortisol levels and aggression. The discrepancies may be attributed to various factors, including methodological differences, individual variability in stress reactivity, and the complex interplay of other neurochemical systems involved in aggression.

### Impact of chronic stress on the HPA axis and aggressive behavior

Chronic stress has a profound impact on the hypothalamic-pituitary-adrenal (HPA) axis and is closely linked to the expression of aggressive behavior. One of the key consequences of chronic stress on the HPA axis is the dysregulation of cortisol, the primary glucocorticoid hormone released during stress. Cortisol plays a crucial role in modulating various physiological processes, including immune function, metabolism, and cognition. However, under chronic stress conditions, cortisol levels may become dysregulated, leading to either hypersecretion or blunted cortisol responses. Both scenarios can have significant implications for aggressive behavior.

Hyperactivation of the HPA axis, resulting in elevated cortisol levels, has been associated with increased aggression. It is suggested that chronic elevated cortisol levels in blood decreases the sensitivity of the hypothalamus to cortisol thereby inhibiting it from exerting its negative feedback mechanism (Gjerstad et al. 2018). The desensitized hypothalamus loses its ability to coordinate incoming information from the limbic system, reticular areas, thalamus, amygdala, and hippocampus (Calderon et al. 2016). This loss of control creates exaggerated neurochemical, emotional and physical responses within the sympathetic nervous system (SNS) that further dysregulates the HPA axis, leading the behavioral changes such as aggression (Herman et al. 2016). Heightened cortisol levels have been shown to have direct effects on brain regions involved in the regulation of emotions, such as the amygdala and prefrontal cortex (Veer et al. 2012). The amygdala plays a crucial role in processing and generating emotional responses, including fear and aggression. Increased cortisol levels can enhance the amygdala’s reactivity to threat cues, leading to heightened emotional responses and a propensity for aggressive behavior (Hakamata et al. 2017). At the same time, chronic stress-induced cortisol elevation may impair the prefrontal cortex’s executive functions, which are important for regulating impulsive and aggressive behaviors. This disruption in prefrontal cortical control can further contribute to the expression of aggressive tendencies.

On the other hand, chronic stress can also result in HPA axis dysregulation characterized by blunted cortisol responses, often referred to as hypocortisolism. Hypocortisolism has been observed in individuals with a history of chronic stress, such as victims of childhood maltreatment or individuals with post-traumatic stress disorder (PTSD) (Siever 2008). This dysregulation is thought to be a result of prolonged exposure to stress hormones, leading to a downregulation of the HPA axis. Interestingly, blunted cortisol responses have also been associated with increased aggression. The exact mechanisms underlying this relationship are not fully understood, but it is believed that alterations in the stress response system can lead to compensatory mechanisms, such as increased activation of the sympathetic nervous system and alterations in other neurochemical systems, which can contribute to the expression of aggression.

Furthermore, chronic stress-induced changes in the HPA axis also have long-lasting effects on brain structures involved in the regulation of aggressive behavior. The hippocampus, a region critical for memory and emotion regulation, is highly susceptible to the impact of chronic stress. Chronic stress has been shown to induce structural changes in the hippocampus, including dendritic remodeling, reduced neurogenesis, and decreased hippocampal volume. These alterations in hippocampal structure and function may disrupt the balance between emotional regulation and cognitive control, leading to an increased risk of aggressive behavior (Šimić et al. 2021).

Chronic stress exerts a significant impact on the HPA axis, leading to dysregulation of cortisol levels and altering the neurobiology underlying aggressive behavior. Both hyperactivation and blunted cortisol responses have been associated with increased aggression, likely through their effects on brain regions involved in emotional processing and cognitive control (Ghasemi et al. 2021; Murphy et al. 2022). Understanding the complex interplay between chronic stress, the HPA axis, and aggressive behavior is crucial for developing effective interventions to mitigate the negative consequences of chronic stress and promote healthier behavioral outcomes.

## The HPG axis: physiology and implications in aggression

### HPG axis components and functioning

The hypothalamic-pituitary-gonadal (HPG) axis is a crucial neuroendocrine system responsible for regulating reproductive function and the production of sex hormones. It consists of three main components: the hypothalamus, the anterior pituitary gland, and the gonads (testes in males and ovaries in females). Each component plays a vital role in the functioning of the HPG axis. The hypothalamus serves as the primary control center for the HPG axis. Within the hypothalamus, specialized neurons called gonadotropin-releasing hormone (GnRH) neurons release GnRH into the bloodstream. GnRH acts on the anterior pituitary gland, specifically on gonadotroph cells, to regulate the synthesis and release of two key gonadotropins: follicle-stimulating hormone (FSH) and luteinizing hormone (LH). FSH and LH are essential for the functioning of the gonads and the production of sex hormones. The anterior pituitary gland, located at the base of the brain, responds to GnRH by releasing FSH and LH into the bloodstream. FSH acts on the gonads to stimulate the development and maturation of follicles in females and the production of sperm in males. LH, on the other hand, plays a crucial role in triggering ovulation in females and the production of testosterone in males. The release of FSH and LH from the anterior pituitary is tightly regulated by the pulsatile release of GnRH from the hypothalamus (Hiller-Sturmhöfel and Bartke 1998). The gonads, which are the testes in males and the ovaries in females, are responsible for the production of sex hormones.

In males, the Leydig cells within the testes produce testosterone under the influence of LH. Testosterone is the primary male sex hormone and is involved in the development of secondary sexual characteristics, such as facial hair growth and deepening of the voice, as well as the regulation of sexual desire and reproductive function. In females, the ovaries produce estrogen and progesterone in response to FSH and LH. Estrogen and progesterone are critical for regulating the menstrual cycle, promoting the development of secondary sexual characteristics, and maintaining pregnancy.

The functioning of the HPG axis is tightly regulated through a complex feedback system. The sex hormones produced by the gonads exert negative feedback on the hypothalamus and the anterior pituitary gland to regulate their own production. When sex hormone levels are low, the hypothalamus releases more GnRH, which stimulates the anterior pituitary to produce and release FSH and LH. In turn, FSH and LH act on the gonads to promote the synthesis and release of sex hormones. As sex hormone levels increase, they suppress the release of GnRH from the hypothalamus and the secretion of FSH and LH from the anterior pituitary, creating a feedback loop that maintains hormone balance. The HPG axis is crucial for the development and maintenance of reproductive function in both males and females. Disruptions in the HPG axis can lead to a variety of reproductive disorders, such as infertility, menstrual irregularities, and hormonal imbalances (Toufexis et al. 2014). Additionally, alterations in the HPG axis can have broader implications for overall health and well-being, as sex hormones play a role in various physiological processes beyond reproduction, including bone health, cardiovascular function, and mood regulation.

### Testosterone and aggression: animal and human studies

Testosterone, a hormone primarily produced in the testes in males and to a lesser extent in the ovaries in females, has long been implicated in the modulation of aggression. Animal and human studies have provided valuable insights into the relationship between testosterone and aggressive behavior. Research in male mice has shown that perinatal castration, which involves removing the gonads at birth, leads to a reduction in aggressiveness in adulthood (Edwards 1969). On the other hand, in male mice with intact gonads, chronic administration of propionate testosterone in adulthood has limited behavioral effects, primarily reducing the latency of menacing the opponent (Martínez-Sanchis et al. 1998). Interestingly, studies have also investigated the role of androgens in female mice selected for intraspecific aggressiveness (TA) compared to non-aggressive females (TNA). In TA females, neonatal androgenization led to increased aggression, like what is observed in TA males. However, non-androgenized TA females and TNA females did not display aggression (French et al. 2013). These findings suggest that androgens can affect aggressive behavior in the presence of specific genetic backgrounds, although genetic predisposition alone is insufficient to manifest aggressiveness.

More studies have explored the effects of androgens on female mice belonging to different stocks selected for varying levels of aggressiveness. Female mice androgenized at birth from stocks selected for higher aggressiveness levels (SAL: short attack latency) exhibited increased aggression after castration and testosterone administration in adulthood. In comparison, similarly, treated females from stocks selected for lower aggressiveness levels (LAL: long attack latency) showed a less pronounced effect on aggression. Notably, even after castration, the administration of estradiol, a form of estrogen, did not fully reverse the increased aggressiveness in SAL females (Sandnabba et al. 1994). Studies on monkeys, specifically Macaca mulatta, have provided insights into the relationship between testosterone and aggression. In male monkeys, there is a correlation between unstored, free testosterone levels in the cerebrospinal fluid and aggressiveness. During the mating season, an increase in testosterone levels in both the cerebrospinal fluid and plasma has been observed, along with heightened aggressive attitudes (Higham et al. 2013). This link between plasma testosterone levels and aggressiveness has been confirmed in males of the same species. Another study in Macaca fascicularis males demonstrated that testosterone administration not only increased aggressiveness but also enhanced dominance attitudes, particularly among individuals who were already dominant (Rejeski et al. 1988). However, the differentiation between later aggressiveness and dominance poses a question. Dominance can be seen as the attained social level, and this issue becomes more evident in humans (Higham et al. 2013). In a species of lemur, Microcebus murinus, where dominance relationships are established at the beginning of each mating season, older males are always dominant, despite having lower plasma testosterone levels and displaying less aggressive behaviors (Aujard and Perret 1998; Chaudron et al. 2021). In Macaca fascicularis males, when they were placed separately and then in a group, the testosterone and cortisol levels obtained during their separation did not correlate with the social level they achieved within the group (Czoty et al. 2009). However, it is important to note that this study did not observe hormonal levels after interaction with other males, preventing the establishment of whether changes occurred due to the new environmental situation.

Perinatal exposure to testosterone also influences adult behavior in monkeys. Young male monkeys typically engage in more violent play compared to females. If testosterone is administered to mothers during pregnancy, female offspring may develop male genitals and exhibit behaviors like males. Similarly, if testosterone administration occurs later in pregnancy, female offspring will have female genitals, but their behavior will still resemble that of males (Goy et al. 1988). These findings indicate that behavioral attitudes are more sensitive to the presence of testosterone than to genital morphogenesis alone. When it comes to studying the link between testosterone and aggressiveness in adult men, research is limited due to practical and ethical considerations. However, studies have been conducted on individuals with marked aggressive behaviors, such as prisoners, or on individuals using anabolic steroids to enhance muscle mass. In studies involving prisoners, it has been consistently found that those guilty of violent crimes have significantly higher testosterone levels compared to those guilty of other types of crimes (Beit-Hallahmi 1971; Kreuz and Rose 1972). Similarly, prisoners who frequently broke prison rules were found to have higher testosterone levels than those with lower levels (Dabbs et al. 1987). These findings suggest a correlation between elevated testosterone levels and aggressive behaviors in this population. Furthermore, research conducted on a sample of over 4500 military men revealed that plasma testosterone levels were associated with various antisocial behaviors (Dabbs and Morris 1990).

More results from several human studies despite riddled with several limitations either in methodology, small sample size or non-randomized, show that reducing androgens through use of antiandrogens or castration reduce aggression among known sexual offenders (Beech and Mitchell 2005). Findings of a study by Rada et al. (1976) to determine testosterone levels in the plasma of 52 offenders convicted of rape at a California facility for mentally disordered offenders suggested that sexual aggression (rape in this case) probably is not determined largely by high levels of testosterone. These offenders were also screened of alcoholism. Their findings also showed and association between aggression and drinking. Criminals with troubled personalities, particularly habitual criminals, have been found to have higher total and free plasma testosterone levels compared to schizophrenic criminals. Additionally, individuals with a history of frequent impulsive behaviors, alcoholism, and suicide attempts have been found to have high levels of free testosterone in the cerebrospinal fluid. Moreover, there is a direct relationship between plasma testosterone levels and aggressiveness among individuals with alcoholism (Fritz et al. 2023). Given, that alcohol affects plasma testosterone levels, the relationship between testosterone levels and sexual aggression becomes even more confusing in alcoholics.

Despite this uncertainty both surgical and chemical castration undoubtedly reduce sexual interest, sexual performance, and sexual reoffending (Lee and Cho 2013). Compared to non-sexual offenders, Wong and Gravel (2018) found that testosterone levels were higher in sexual offenders. Their findings correlated with prior sexual aggression in the sex offenders. What was not clear was whether the sexual aggression cause high testosterone levels or the high testosterone levels caused the sexual aggression. Studer et al. (2005) however were able to show statistically significant association between serum testosterone levels and subsequent sexual offending in a sample of 501 adult male sex offenders.

While these findings provide evidence of a link between testosterone levels and aggressive behaviors in adult men, it is important to note that these researches focused on individuals with specific characteristics, such as prisoners and individuals with troubled personalities or alcoholism. The generalizability of these findings to the broader population should be interpreted with caution. It is important to note that the relationship between testosterone and aggression is not deterministic, and testosterone alone does not determine aggressive behavior. Aggression is a complex behavior influenced by a combination of biological, genetic, environmental, and social factors. Testosterone may interact with other factors to influence aggressive behavior. For example, social and situational factors, such as provocation, competition, and dominance hierarchies, can modulate the relationship between testosterone and aggression (Knight et al. 2022; Muñoz-Reyes et al. 2020). Furthermore, testosterone can influence aggression indirectly through its effects on other psychological and physiological processes. Testosterone has been found to influence mood, self-esteem, and dominance-seeking behaviors, which can in turn impact aggressive tendencies (Zitzmann 2020). Moreover, testosterone can influence brain regions involved in the regulation of emotion and aggression, such as the amygdala and prefrontal cortex.

### Androgen receptors and neural circuitry involved in aggression

Androgen receptors play a crucial role in modulating aggression by mediating the effects of androgen hormones, such as testosterone, on the brain. Understanding the neural circuitry underlying aggression and the involvement of androgen receptors is essential for unraveling the mechanisms that regulate this behavior. Androgen receptors are expressed in various regions of the brain, including the amygdala, hypothalamus, prefrontal cortex, and striatum, which are known to be involved in aggression.

The amygdala, particularly the basolateral amygdala (BLA), has been implicated in the emotional processing and expression of aggression. Androgen receptors in the BLA are thought to contribute to the facilitation of aggressive behaviors by modulating the excitability and synaptic plasticity of amygdala neurons (Coolen et al. 2021). The hypothalamus, specifically the ventromedial hypothalamus (VMH), is another critical region involved in aggression. Androgen receptors in the VMH have been shown to regulate both offensive and defensive aggression. Activation of androgen receptors in the VMH promotes offensive aggression, while their inhibition reduces aggressive behavior. The VMH acts as a central hub that integrates sensory information and hormonal signals to coordinate aggressive responses (Balfour et al. 2003).

The prefrontal cortex (PFC), particularly the medial prefrontal cortex (mPFC), is involved in the control and regulation of aggression. Androgen receptors in the mPFC are thought to play a role in inhibiting aggressive behavior by modulating executive functions and impulse control. Dysfunction in the PFC, including alterations in androgen receptor signaling, has been associated with increased impulsivity and aggression (Fernàndez-Castillo and Cormand 2016). The striatum, a region involved in reward processing and motor control, also plays a role in aggression. Androgen receptors in the striatum modulate the motivational and rewarding aspects of aggression. Activation of these receptors enhances the reinforcing properties of aggressive behavior, potentially contributing to its persistence (Bialy et al. 2019).

Furthermore, androgen receptors are involved in the modulation of neurotransmitter systems implicated in aggression, such as the serotonin and dopamine systems. Testosterone, through its action on androgen receptors, influences the release and activity of these neurotransmitters, which can impact aggression. For example, testosterone can increase dopamine release in reward-related brain regions, enhancing the motivation for aggressive behavior (Van Erp and Miczek 2000). The intricate interplay between androgen receptors and the neural circuitry involved in aggression highlights the complex nature of this behavior. Dysregulation of androgen receptor signaling or alterations in the connectivity within the aggression-related circuitry can contribute to abnormal aggression seen in various neuropsychiatric disorders.

## Interactions between the HPA axis and the HPG axis

### Feedback mechanisms and crosstalk between axes

The hypothalamic-pituitary-adrenal (HPA) axis and the hypothalamic-pituitary-gonadal (HPG) axis are two important neuroendocrine systems that interact with each other through feedback mechanisms and crosstalk. These interactions are crucial for maintaining hormonal balance and coordinating physiological processes related to stress, reproduction, and behavior. The HPA axis is primarily involved in the stress response, with the hypothalamus releasing corticotropin-releasing hormone (CRH), which stimulates the anterior pituitary gland to secrete adrenocorticotropic hormone (ACTH). ACTH then acts on the adrenal glands, leading to the release of glucocorticoid hormones, such as cortisol. These glucocorticoids exert negative feedback on the HPA axis by inhibiting the release of CRH and ACTH, thereby reducing the production of cortisol. The HPG axis, on the other hand, regulates reproductive function. The hypothalamus releases gonadotropin-releasing hormone (GnRH), which stimulates the anterior pituitary gland to secrete follicle-stimulating hormone (FSH) and luteinizing hormone (LH). FSH and LH act on the gonads (ovaries in females and testes in males) to regulate the production of sex hormones, including estrogen and progesterone in females, and testosterone in males. These sex hormones in turn provide feedback to the hypothalamus and pituitary gland to regulate the release of GnRH, FSH, and LH (Heck and Handa 2019).

The HPA and HPG axes are pathways that exist side by side, with both undergoing neuroendocrine changes that seem to begin and end with the hypothalamus in a feedback system manner (Dismukes et al. 2015). The structural components that make up both axes are in the central nervous system and peripheral tissues and include the hypothalamus, anterior pituitary gland, the gonads, and the adrenal glands (Sheng et al. 2021a). Cortisol and testosterone, effectors of the HPA and HPG axes respectively negatively feedbacked on the hypothalamus and the anterior pituitary, which are the same structures in the brain responsible for initiation of the cascade (Papargiris et al. 2011; Pierce et al. 2008). Furthermore, both axes share common regulatory mechanisms. For instance, CRH, which is primarily associated with the HPA axis, has also been found to have effects on the HPG axis. CRH receptors are present in the hypothalamus and pituitary gland, suggesting a direct influence of CRH on the release of GnRH, FSH, and LH. This indicates a potential crosstalk between the two axes at the level of the hypothalamus and pituitary gland. In a pilot study of salivary testosterone and cortisol interrelationships it was found that higher testosterone levels and lower cortisol levels are associated with higher levels of aggression (Brown et al. 2008). Testosterone in the hypothalamus exerts an inhibitory action on CRF and the AVP hormone induces a reduction in cortisol production (Sheng et al. 2021b). More pronounced is the inhibitory effect of cortisol on GnRH (Iwasa et al. 2017). Stressful situations, such as trauma and the like, inflict significant inhibition on testosterone secretion. High testosterone levels or an increase in basal concentrations are associated with aggressive manifestations, whereas high cortisol concentrations are linked to submissive behavior (Batrinos 2012; Deuter et al. 2021).

There are several feedback mechanisms and crosstalk between the HPA and HPG axes. One important aspect is the modulation of gonadal function by stress. During periods of chronic stress, increased levels of cortisol from the HPA axis can impact the HPG axis. High levels of cortisol can suppress the release of GnRH, leading to reduced secretion of FSH and LH, and subsequently affecting reproductive function. This mechanism helps to divert resources away from reproduction and prioritize stress adaptation. Conversely, sex hormones from the HPG axis can also influence the HPA axis. Studies have shown that estrogen, for example, can have a modulating effect on the stress response. Estrogen has been found to enhance the negative feedback of cortisol on the HPA axis, leading to a more efficient regulation of the stress response (Fedotova et al. 2017). Testosterone, on the other hand, has been implicated in the regulation of aggressive behavior, which can have indirect effects on the stress response.

Following exposure to stressful stimuli, the HPA axis is activated with eventual secretion of secretion of glucocorticoid hormones such as cortisol. It is assumed that the secreted glucocorticoids reduce gonadal activity centrally by either reducing pituitary responsiveness to GnRH or inhibiting hypothalamic synthesis and secretion of GnRH (Toufexis et al. 2014). Studies by Iwasa et al. (2017) found that elevated levels of cortisol following stress directly reduced gonadal functions in ovariectomised ewes not by inhibiting GnRH actions but by reducing the responsiveness of the anterior pituitary gland to both endogenous and exogenous GnRH pulses. While previously showing that cortisol infusion reduced the luteinizing hormone (LH) response to fixed hourly GnRH injections in ovariectomized ewes treated with estradiol during the non-breeding season, Pierce et al. (2009) set out to determine if cortisol acts directly at the pituitary or indirectly via the hypothalamus in the presence of oestradiol to inhibit pituitary responsiveness to GnRH in ovariectomised ewes that had undergone surgical hypothalamic–pituitary disconnection. They found that in the absence of estradiol, there was no effect of cortisol on LH pulse amplitude in GnRH-replaced ovariectomized hypothalamic-pituitary disconnected ewes. The also observed that LH pulse amplitude was reduced when cortisol was infused during estradiol treatment. They concluded that the ability of cortisol to reduce LH secretion does not depend upon the frequency of GnRH stimulus and that estradiol enables cortisol to act directly on the pituitary of ovariectomized hypothalamic-pituitary disconnected ewes to suppress the responsiveness to GnRH.

Overall, the feedback mechanisms and crosstalk between the HPA and HPG axes allow for coordinated regulation of stress and reproductive processes. The interactions between these axes help to maintain hormonal balance and adaptability in response to changing environmental and physiological conditions. Disruptions in these feedback mechanisms and crosstalk can have significant implications for health and may contribute to the development of disorders related to stress and reproductive dysfunction. Further research is needed to fully understand the intricate interplay between the HPA and HPG axes and its implications for human physiology and pathology.

### Influence of stress on reproductive functions

Stress has been shown to have significant influence on reproductive functions, affecting various aspects of the hypothalamic-pituitary-gonadal (HPG) axis and ultimately impacting fertility and reproductive health. The HPG axis plays a crucial role in regulating reproductive function in both males and females, and stress-induced disruptions in this axis can have profound effects.

One of the primary ways in which stress affects reproductive function is through the hypothalamus, which is a key regulator of the HPG axis. During periods of stress, the hypothalamus releases corticotropin-releasing hormone (CRH) as part of the stress response. CRH can directly impact the secretion of gonadotropin-releasing hormone (GnRH), which is responsible for initiating the release of follicle-stimulating hormone (FSH) and luteinizing hormone (LH) from the anterior pituitary gland. High levels of CRH can inhibit GnRH release, leading to reduced secretion of FSH and LH, and subsequently impacting ovulation in females and spermatogenesis in males (Maggi et al. 2016).

Stress also disrupts the balance of sex hormones in the body, further influencing reproductive function. In females, chronic stress can lead to disturbances in the menstrual cycle, including irregular or absent periods. This can be attributed to alterations in the production of estrogen and progesterone, which are critical for maintaining reproductive health. Stress-induced hormonal imbalances can interfere with the maturation of ovarian follicles and the release of eggs, thereby reducing fertility (Gallo-Payet et al. 2017). Similarly, stress can affect testosterone production in males. Chronic stress has been associated with decreased testosterone levels, which can impact sperm production and fertility. Testosterone plays a crucial role in spermatogenesis and the maintenance of reproductive function in males. Stress-induced reductions in testosterone can lead to impaired sperm quality, reduced sperm motility, and decreased sperm count, all of which can contribute to infertility. Furthermore, stress-induced changes in the HPG axis also impact sexual behavior and libido. Increased levels of stress hormones, such as cortisol, can suppress sexual desire and impair sexual function. Chronic stress can lead to decreased libido, erectile dysfunction in males, and difficulties with arousal and orgasm in both males and females (Davey and Grossmann 2016).

The mechanisms underlying the influence of stress on reproductive functions are complex and involve interactions between the HPG axis and other systems in the body. Stress hormones can influence the release and action of sex hormones, disrupt the communication between the hypothalamus and pituitary gland, and affect the responsiveness of target tissues to sex hormones. Additionally, stress can induce systemic inflammation and oxidative stress, which can further contribute to reproductive dysfunction. The impact of stress on reproductive functions however can vary among individuals and depend on factors such as the duration and intensity of stress, individual susceptibility, and overall health.

### Role of androgens in stress-induced aggression

Androgens, including testosterone, play a significant role in stress-induced aggression. When individuals experience stressful situations, the release of stress hormones, such as cortisol, triggers a cascade of physiological responses to help cope with the stressor. Among these responses, androgens are known to modulate the behavioral and emotional components of stress, including aggression.

Research has shown that testosterone levels are associated with aggressive behavior, and stress can further enhance this relationship. In both animal and human studies, it has been observed that acute stress can increase testosterone levels, which in turn can influence aggressive responses. Elevated testosterone levels have been linked to increased aggression and dominance-seeking behaviors, especially in the context of social interactions and competition. The effects of testosterone on aggression are mediated through multiple mechanisms. Testosterone can directly influence neural circuits involved in aggression, such as the amygdala, hypothalamus, and prefrontal cortex. These brain regions regulate emotional processing, social behavior, and decision-making, and alterations in their functioning can lead to changes in aggressive responses. Testosterone interacts with androgen receptors in these regions, influencing the neural activity and neurotransmitter systems involved in aggression (Zuloaga et al. 2020). Moreover, testosterone can modulate the reactivity of the hypothalamic-pituitary-adrenal (HPA) axis, the stress-response system in the body. Studies have shown that testosterone can dampen the stress response by reducing the release of cortisol, the primary stress hormone. This modulation of the HPA axis by testosterone may contribute to the regulation of stress-induced aggression (Viau 2002).

It is important to note that the relationship between testosterone and aggression is complex and influenced by various factors. Individual differences, such as genetic predispositions and early life experiences, can impact the sensitivity of individuals to the effects of testosterone on aggression. Contextual factors, such as the nature of the stressor and the social environment, also play a role in shaping the relationship between testosterone and aggression.

Studies have reported that isolated individuals have shrunken brain size especially in the prefrontal cortex, a region important in decision making and social behavior (Tsai 2018). Isolated rodents as well show disrupted signaling in the prefrontal cortex (Bzdok and Dunbar 2020). Data from functional neuroimaging and lesion neuropsychology have demonstrated that the volume of the amygdala correlates positively with social network size and complexities in adult humans indicating that the amygdala is critical in processing emotions and brain networks contributing to social behavior, suggesting that social isolation affects one’s emotions (Liu et al. 2012; Manes et al. 2002).

Stress from prolonged social isolation has also been observed to decrease the size and activities of: (1) the bed nucleus stria terminalis (BNST) which plays a role in sustained fear states, and social attachment behaviors (which comprises aggressive behavior, initiation of mating, offspring and parental bonding); and (2) the lateral septal nucleus (LS) which plays a critical role in emotionality and social behavior, via structural connections with the hypothalamus and hippocampus (Bickart et al. 2012; Kwak et al. 2018). Stress, besides influencing behavior via activating the HPA axis, also affects behavior through activating the mesolimbic dopamine system (Deng et al. 2019; Morinan and Leonard 1980).

The effects of testosterone on aggression are not solely determined by its absolute levels but also by the balance between testosterone and other hormones, such as cortisol and serotonin. Imbalances in these hormonal systems can influence the expression of aggression. There appears to be testosterone/cortisol ratio that influences sexual aggressive behavior. In low testosterone/cortisol ratio, that is in situations of stress, cortisol through the prefrontal cortex, facilitates cognitive control on impulsive tendencies aroused by the emotional subcortical structures. In situations of high testosterone/cortisol ratio occurs it is more likely to result in socially aggressive behavior and sexual aggression (Mehta and Josephs 2010). Cortisol as well as serotonin receptors are also expressed in amygdala neurons. These two neurochemicals act antagonistically to testosterone in the manipulation of subcortical emotional activity and the restraining interference of the prefrontal cortex. At the neuronal level of this hormonal imbalance, testosterone activates emotional processes in the amygdala increasing the resistance of this subcortical structure to prefrontal inhibiting activity.

## The androgen system and aggression

### Testosterone and aggression: causal or correlative relationship?

The relationship between testosterone and aggression has been a topic of extensive research, yet the nature of this relationship remains complex and subject to ongoing debate. While numerous studies have found associations between testosterone levels and aggressive behavior, the question of whether testosterone directly causes aggression or if it simply correlates with aggressive tendencies remains unresolved.

One challenge in determining causality is the bidirectional nature of the testosterone-aggression relationship. On the one hand, there is evidence to suggest that testosterone can influence aggressive behavior. Animal studies have shown that manipulations of testosterone levels, such as castration or testosterone supplementation, can alter aggression levels in various species (Fedotova et al. 2017). Similarly, human studies have found that individuals with higher testosterone levels, whether naturally or through exogenous administration, tend to exhibit more aggressive behaviors (Santi et al. 2020). These findings suggest that testosterone may have a causal role in promoting aggression. On the other hand, it is also plausible that aggression can influence testosterone levels. Aggressive encounters, such as confrontations or competitive situations, have been shown to transiently elevate testosterone levels (Oliveira et al. 2014). This suggests that aggression itself may stimulate testosterone production as part of the physiological stress response. Therefore, it is possible that aggression and testosterone levels have a bidirectional relationship, with each influencing the other.

Additionally, the relationship between testosterone and aggression is influenced by various contextual factors. Social and environmental factors can interact with testosterone to shape aggressive behavior. For example, research has found that testosterone may have a stronger impact on aggression in the presence of provocation or social threat (Munley et al. 2018). This suggests that the effects of testosterone on aggression are contingent on situational factors, and the relationship is not solely determined by testosterone levels alone. Furthermore, individual differences and other biological and psychological factors may moderate the testosterone-aggression relationship. Genetic factors, prenatal hormone exposure, and early life experiences can influence an individual’s sensitivity to the effects of testosterone on aggression (Clark et al. 2018). Psychological factors, such as personality traits and self-control, also interact with testosterone to shape aggressive tendencies. These factors highlight the complex interplay between biology and environment in determining aggressive behavior.

The relationship between testosterone and aggression is multifaceted, and establishing a causal or correlative link between the two is challenging. While there is evidence to suggest that testosterone can influence aggressive behavior, the bidirectional nature of this relationship, as well as the influence of contextual and individual factors, complicate the understanding of causality. Further research is needed to unravel the underlying mechanisms and determine the precise nature of the testosterone-aggression relationship. Such investigations may involve experimental manipulations of testosterone levels, longitudinal studies, and sophisticated statistical analyses to disentangle the complex interactions between testosterone, aggression, and other contributing factors.

### Neural correlates of androgen modulation of aggressive behavior

Understanding the neural correlates of androgen modulation of aggressive behavior is crucial for unraveling the underlying mechanisms that link testosterone to aggression. Research has shed light on several brain regions and neural pathways involved in this process. One key region implicated in the androgen modulation of aggression is the amygdala, particularly its basolateral complex. The amygdala plays a crucial role in processing emotional stimuli and generating appropriate behavioral responses, including aggression. Androgen receptors are highly expressed in the amygdala, and testosterone can modulate amygdala activity (Cunningham et al. 2012). Testosterone has been associated with changes in the activity of the amygdala, orbitofrontal cortex (OFC), and insula as well as a reduction in the functional amygdala/prefrontal connection (van Wingen et al. 2010). Little is known about the links between these associations and long-term testosterone secretion because such associations have only previously been explored using acute measures of testosterone. Animal studies have shown that testosterone can enhance amygdala responsiveness to social and threat-related cues, thereby promoting aggressive behavior. Moreover, testosterone’s effects on the amygdala may be mediated by interactions with other neurotransmitter systems, such as serotonin and GABA, which can influence aggression (Montoya et al. 2012).

Another important brain region involved in androgen modulation of aggression is the prefrontal cortex (PFC). The PFC is responsible for executive functions, impulse control, and decision-making, and it plays a crucial role in inhibiting inappropriate or impulsive aggressive responses. Testosterone has been shown to affect PFC activity and connectivity, with higher testosterone levels associated with decreased PFC activation and reduced inhibitory control (Volman et al. 2016). This may lead to a disinhibition of aggressive behavior. Furthermore, the PFC and amygdala are connected by reciprocal pathways, and the balance between their interactions may determine the expression of aggression.

The hypothalamus, a critical regulator of the endocrine system, also plays a role in androgen modulation of aggression. The hypothalamus contains clusters of neurons that produce neuropeptides, such as vasopressin and oxytocin, which have been implicated in social and aggressive behaviors. Testosterone influences the production and release of these neuropeptides, thereby modulating aggression. For example, vasopressin has been linked to territorial aggression and social dominance, while oxytocin has been associated with social bonding and affiliative behaviors (Albers 2012; Rigney et al. 2022). Testosterone interacts with these neuropeptides to shape the neural circuitry underlying aggressive behavior. Estradiol and testosterone (T) control the expression of arginine-vasopressin (AVP) in various neuronal populations through unclear processes. It has been demonstrated that estrogen receptors (ERs) take involvement in estradiol’s modulation of AVP neurons (Sladek and Somponpun 2008). Additionally, there is evidence that Testosterone’s metabolite 5’-dihydrotestosterone (5’-DHT) and subsequent conversion into the androgen metabolite and ER ligand 3’-diol are exerting Testosterone’s control over AVP expression (Handa et al. 2009).

Furthermore, the mesolimbic dopamine system, which includes the ventral tegmental area (VTA) and the nucleus accumbens (NAcc), has been implicated in the androgen modulation of aggression. Testosterone enhances dopamine release in the NAcc, which is involved in reward processing and motivation. Testosterone boosts motivation and reward processing, which raises the possibility of behavioral instability. This dopaminergic system may reinforce and strengthen aggressive behaviors, contributing to their persistence (Chester et al. 2016). The neural correlates of androgen modulation of aggressive behavior involve several interconnected brain regions and neural pathways. The amygdala, PFC, hypothalamus, and mesolimbic dopamine system play crucial roles in mediating the effects of testosterone on aggression. These regions interact with each other and with other neurotransmitter systems to shape the neural circuitry underlying aggressive behavior. Further research is needed to better understand the precise mechanisms by which testosterone influences these neural correlates and how they interact with other factors, such as genetics, environmental influences, and individual differences, to determine the expression of aggressive behavior.

### Androgens and social stress: implications for aggressive phenotypes

Androgens, including testosterone, play a crucial role in the regulation of social behavior and the response to social stressors, which can have significant implications for the development of aggressive phenotypes. Research has shown that androgens modulate social behavior by influencing various cognitive and emotional processes involved in social interactions. Moreover, androgens have been found to interact with stress-responsive systems, such as the hypothalamic-pituitary-adrenal (HPA) axis, to shape behavioral responses to social stress. Animal studies have shown that manipulations of androgen levels, either through pharmacological interventions or genetic modifications, can lead to alterations in aggressive behavior. In a study to determine whether prenatal testosterone exposure could affect the body weight, anogenital distance, anogenital distance index, puberty onset, social behavior, fertility, sexual behavior, sexual preference, and Testosterone level of male rats in adulthood, it was discovered that Testosterone exposure could change significant facets of sexual and social behavior even though these animals were effective at producing offspring (Hines et al. 2015). Other studies have shown that, castration or anti-androgen treatments in male animals reduce aggression, while exogenous administration of androgens can restore or increase aggressive behavior (Cunningham et al. 2012). These findings highlight the causal relationship between androgens and aggressive phenotypes. In humans, correlational studies have consistently shown positive associations between circulating testosterone levels and aggressive behavior, particularly in males (Carré and Archer 2018). Research has focused on understanding the role of androgens in the context of social stress, as stressful social situations often elicit aggressive responses. It has been observed that exposure to social stressors can lead to an acute increase in testosterone levels, which may facilitate aggressive behavior as a means of coping with the stressor. However, the relationship between androgens and aggression in humans is complex and multifaceted, influenced by individual differences, contextual factors, and the interplay of various biological and psychological mechanisms.

Furthermore, androgens interact with the HPA axis, which is a major stress-responsive system involved in the regulation of physiological and behavioral responses to stress. Studies have shown that androgens can modulate HPA axis activity, with evidence suggesting that testosterone may act as a negative regulator of the stress response. Testosterone has been found to attenuate cortisol responses to stress, potentially reducing the impact of stress on aggressive behavior. Conversely, chronic stress and dysregulation of the HPA axis can influence androgen levels and contribute to the development of aggressive phenotypes. Understanding the interplay between androgens, social stress, and aggressive behavior has important implications for interventions targeting aggression and related disorders. Pharmacological manipulations of androgen levels, such as androgen receptor blockers or hormone replacement therapies, have been explored as potential treatment approaches for aggression-related conditions. Androgens, particularly testosterone, are important regulators of social behavior and play a role in shaping aggressive phenotypes. The interaction between androgens, social stress, and the HPA axis provides insight into the mechanisms underlying aggressive behavior. Further research is needed to elucidate the complex interplay between androgens, social stress, and aggression, which can inform the development of targeted interventions for aggression-related conditions.

## Future directions and implications for clinical interventions

### Unraveling the neurobiological mechanisms: integrating molecular, cellular, and circuit-level approaches

Understanding the neurobiological mechanisms underlying aggression requires a comprehensive investigation that integrates molecular, cellular, and circuit-level approaches. Aggressive behavior is a complex phenomenon influenced by a network of interconnected brain regions and intricate molecular signaling pathways. By combining multiple levels of analysis, researchers can gain insights into the underlying mechanisms that drive aggression. At the molecular level, studies have focused on identifying specific genes and their associated signaling pathways that contribute to aggressive behavior. Genetic studies in both animals and humans have identified candidate genes that are involved in neurotransmitter regulation, hormone signaling, and synaptic plasticity. In human studies, various genetic variants have been implicated in aggression. For example, polymorphisms in genes encoding the serotonin transporter (5-HTT), monoamine oxidase A (MAOA), and dopamine receptors have been associated with increased risk of aggressive behavior (Cahill et al. 2022; Fernàndez-Castillo and Cormand 2016). These variants can influence neurotransmitter levels and the functioning of brain regions involved in emotional regulation and impulse control. Additionally, genes involved in the regulation of the hypothalamic-pituitary-adrenal (HPA) axis, such as the glucocorticoid receptor gene (NR3C1), have been linked to aggression, highlighting the role of stress response systems in aggressive phenotypes Liu et al. 2021; Mbiydzenyuy et al. 2022). Animal studies have provided valuable insights into the genetic basis of aggression. Through selective breeding, researchers have identified specific gene variants associated with aggressive behavior in various species, including rodents and primates. For instance, studies in mice have identified genes involved in the regulation of the serotonin system, such as Tph2 and Maoa, as key players in modulating aggression (Mark et al. 2019; Takahashi et al. 2012). Similarly, in rhesus macaques, genetic variants in the serotonin transporter gene (SLC6A4) have been associated with individual differences in aggressive behavior (Lindell et al. 2012; Madrid et al. 2018). These genes can influence the development and function of neural circuits implicated in aggression. Molecular approaches such as gene expression profiling, epigenetic modifications, and gene knockout models provide valuable tools to investigate the molecular basis of aggression.

Moving to the cellular level, researchers investigate the role of specific cell types and their interactions in aggression. Different types of neurons, including excitatory and inhibitory neurons, may play distinct roles in shaping aggressive behavior. Neuronal activity and connectivity within specific brain regions, such as the amygdala, prefrontal cortex, and hypothalamus, are critical for the expression and modulation of aggression. In human studies, neuroimaging techniques such as functional magnetic resonance imaging (fMRI) have been utilized to identify brain regions associated with aggressive behavior. The amygdala, prefrontal cortex (PFC), and anterior cingulate cortex (ACC) have emerged as key players in aggression. Increased amygdala activation has been observed during aggressive episodes, indicating its involvement in the processing of emotional stimuli and the generation of aggressive responses. The PFC, particularly the ventromedial PFC (vmPFC), is responsible for inhibitory control and decision-making processes, and disruptions in this region have been linked to impulsive and aggressive behavior. The ACC is involved in monitoring and regulating emotional responses and has been implicated in aggression-related deficits in empathy and self-control (Bounoua et al. 2022). Animal studies have complemented human research by providing a more detailed understanding of the neural circuitry underlying aggression. Studies in rodents have identified specific brain regions, such as the hypothalamus, periaqueductal gray, and lateral septum, as key nodes in the aggression network. Manipulations of neuronal activity within these regions have been shown to modulate aggressive behavior. Additionally, research in non-human primates has highlighted the role of the amygdala, PFC, and striatum in the regulation of aggression, mirroring findings in humans. Moreover, studies have investigated the connectivity between these brain regions to elucidate the functional networks involved in aggression. Disruptions in the connectivity between the amygdala and PFC, for example, have been associated with aggression-related traits. Animal studies utilizing techniques like optogenetics and chemogenetics have provided causal evidence for the involvement of specific circuits in aggression. Advanced techniques such as optogenetics and calcium imaging allow researchers to manipulate and monitor the activity of specific cell populations in real-time, providing insights into the cellular mechanisms underlying aggression.

To understand how these molecular and cellular processes interact within neural circuits, circuit-level approaches are employed. Neural circuits comprise interconnected populations of neurons that work together to process information and generate behavior. Mapping and manipulating these circuits can uncover the precise circuitry involved in aggression. Techniques such as circuit tracing, functional connectivity mapping, and circuit-specific manipulations, such as chemogenetics and electrical stimulation, enable researchers to dissect the functional organization of aggression-related circuits. Integration of these multiple levels of analysis is crucial for unraveling the neurobiological mechanisms of aggression. By examining how molecular changes influence cellular properties, and how these cellular changes impact circuit activity and behavior, a more comprehensive understanding of aggression can be achieved. Moreover, these integrated approaches allow researchers to identify potential targets for therapeutic interventions aimed at mitigating aggression-related disorders. It is worth noting that aggression is a complex behavior influenced by various factors, including genetic predisposition, environmental factors, and social context. Therefore, studying the neurobiological mechanisms of aggression requires a multi-dimensional approach that considers the dynamic interactions between genes, cells, and circuits within the broader social and environmental context. Unraveling the neurobiological mechanisms of aggression necessitates an integrated approach that combines molecular, cellular, and circuit-level investigations. By examining the molecular and cellular processes underlying aggression and their interactions within neural circuits, researchers can gain valuable insights into the complex nature of aggression. This interdisciplinary approach not only enhances our understanding of aggression but also opens new avenues for the development of targeted interventions for aggression-related disorders.

### Translational perspectives: from animal models to Human studies

Translating findings from animal models to human studies is a critical step in understanding the neurobiological basis of aggression and developing effective interventions. Animal models provide a controlled environment where researchers can manipulate genetic, neurochemical, and environmental factors to study the underlying mechanisms of aggression. These models allow for the identification of potential targets for intervention and the testing of novel therapeutic strategies. Animal models have revealed important insights into the neural circuits and molecular pathways involved in aggression. By selectively manipulating specific genes or brain regions in animals, researchers can observe the effects on aggressive behavior. For example, studies using knockout, or overexpression models have demonstrated the role of genes such as the androgen receptor, serotonin transporter, and neuropeptides in modulating aggression (Takahashi and Miczek 2014). Additionally, studies utilizing techniques like optogenetics and chemogenetics have allowed for the precise control of neural activity and the identification of circuit-level mechanisms underlying aggression.

Translating these findings to human studies is essential for validating the relevance of animal models and understanding the applicability of the findings to human behavior. Human studies provide an opportunity to examine the genetic and neurobiological factors associated with aggression in a more direct manner. Neuroimaging techniques, such as functional magnetic resonance imaging (fMRI) and positron emission tomography (PET), enable researchers to investigate the brain activity and connectivity patterns in individuals exhibiting aggressive behavior. These studies have implicated brain regions such as the amygdala, prefrontal cortex, and anterior cingulate cortex in aggression. In addition to neuroimaging, genetic studies in humans have identified genetic variants associated with aggression. Genome-wide association studies (GWAS) and candidate gene approaches have identified genetic polymorphisms related to neurotransmitter systems, hormone signaling, and neurodevelopmental processes (Ip et al. 2021). These genetic findings provide valuable insights into the molecular basis of aggression in humans.

Furthermore, clinical studies involving individuals with aggression-related disorders, such as conduct disorder, antisocial personality disorder, and intermittent explosive disorder, provide important information about the manifestation and treatment of aggression in real-world contexts. These studies assess the effectiveness of pharmacological interventions, psychotherapy, and behavioral interventions in reducing aggressive behavior. They also shed light on the influence of environmental factors, such as childhood adversity and social context, on aggression. Overall, integrating findings from animal models with human studies offers a comprehensive understanding of the neurobiological underpinnings of aggression. Animal models provide a controlled experimental platform to investigate molecular and circuit-level mechanisms, while human studies validate these findings and explore their relevance in real-world settings. This translational approach facilitates the development of targeted interventions and therapeutic strategies for individuals with aggression-related disorders. By bridging the gap between animal and human research, we can advance our knowledge of aggression and ultimately improve the lives of those affected by aggressive behaviors.

### Pharmacotherapeutic strategies for aggression-related disorders

Aggression can be associated with various psychiatric conditions, including but not limited to, disruptive behavior disorders, mood disorders, personality disorders, and certain neurodevelopmental disorders. The use of medications aims to reduce aggression, improve impulse control, and promote emotional regulation. One class of medications commonly used in the treatment of aggression-related disorders is antipsychotics. These medications, such as risperidone and olanzapine, target the dopamine receptors in the brain. By blocking dopamine activity, antipsychotics help regulate neurotransmitter imbalances and stabilize mood. They are frequently prescribed for conditions like schizophrenia, bipolar disorder, and conduct disorder, where aggression is a prominent symptom (Tseligkaridou et al. 2023).

Mood stabilizers are another pharmacotherapeutic approach utilized in managing aggression-related disorders. Medications like lithium and valproate are often prescribed for individuals with bipolar disorder. These agents help stabilize mood swings and impulsive behaviors, which can contribute to aggressive outbursts. By modulating neurotransmitter activity and promoting emotional stability, mood stabilizers can effectively reduce aggression. Selective serotonin reuptake inhibitors (SSRIs) are commonly used in the treatment of aggression associated with depressive disorders, anxiety disorders, and certain personality disorders. Medications such as fluoxetine and sertraline increase serotonin levels in the brain by inhibiting its reuptake, thereby improving mood and reducing impulsive and aggressive behaviors. SSRIs are valuable in addressing the underlying emotional dysregulation that may contribute to aggression (O’donnell et al. 2022).

In acute situations where immediate intervention is necessary to manage aggression and agitation, benzodiazepines can be employed. Medications like diazepam and lorazepam have sedative and calming effects, helping to alleviate acute episodes of aggression (Zaman et al. 2017). They act on the gamma-aminobutyric acid (GABA) receptors in the brain, which are involved in reducing excitability and promoting relaxation. For individuals with attention-deficit/hyperactivity disorder (ADHD) or conduct disorder, alpha-2 adrenergic agonists like clonidine and guanfacine can be beneficial in managing aggression (Mechler et al. 2022). These medications regulate norepinephrine levels in the brain and improve impulse control. By targeting the alpha-2 adrenergic receptors, they help reduce hyperactivity, impulsivity, and aggressive behaviors often associated with these disorders.

## Conclusion

This comprehensive review highlights the intricate relationships between stress, the HPA axis, the HPG axis, and the androgen system in the context of aggression. The findings underscore the importance of multidisciplinary investigations that integrate neuroscience, endocrinology, and behavioral sciences to unravel the complex neuroendocrine mechanisms underlying aggression. Further research is needed to elucidate the causal relationships between stress, hormonal regulation, and aggressive behavior, as well as to identify novel therapeutic targets for aggression-related disorders. Understanding the interplay between these systems holds significant promise for advancing our knowledge and developing effective interventions in the field of aggression research.

## Data Availability

Not applicable.
